# Changes in Gene Expression Patterns in Young and Senescent Fibroblasts in Glycated Three-Dimensional Collagen Matrices

**DOI:** 10.3390/ijms26104769

**Published:** 2025-05-16

**Authors:** Zulfiya G. Guvatova, Evelina R. Kudasheva, Yuri M. Efremov, Peter S. Timashev, Maria S. Fedorova, Elena A. Pudova, Anastasiya V. Snezhkina, Anna V. Kudryavtseva, Anastasiya A. Kobelyatskaya, Alexey A. Moskalev

**Affiliations:** 1 Engelhardt Institute of Molecular Biology, Russian Academy of Sciences, 119991 Moscow, Russia; 2 Russian Clinical Research Center for Gerontology, Pirogov Russian National Research Medical University, Ministry of Healthcare of the Russian Federation, 127994 Moscow, Russia; 3 Institute for Regenerative Medicine, Sechenov First Moscow State Medical University (Sechenov University), 119991 Moscow, Russia; 4 Longevity Institute of Russian Research Surgery Center, 119435 Moscow, Russia

**Keywords:** glycation, senescence, ECM stiffness, D-ribose, gene expression, transcriptome

## Abstract

Glycation, or non-enzymatic glycosylation, has recently attracted increasing interest in the context of its impact on aging. Advanced glycation end products (AGEs) contribute to various age-related pathological conditions such as inflammation, fibrosis, and vascular calcification. However, the molecular mechanisms underlying glycation-induced disruption of cell–matrix interactions during cellular senescence are not fully understood. The aim of this study was to investigate transcriptomic changes in young and senescent dermal fibroblasts (HdFbs) cultured in 3D post-glycated collagen type I matrices after 10 and 17 days. Our findings indicate that D-ribose-mediated glycation increases the accumulation of fluorescent AGEs and the stiffness of matrices in a dose-dependent manner. The transcriptome alterations in cells encompassed the modulation of age-related genes and signaling pathways, including activation of genes related to senescence-associated secretory phenotype (SASP). Notably, the alterations in the transcriptome profiles due to glycation were more pronounced (in terms of both the number of genes and their fold changes) after 10 days of culture compared to day 17 in both passages. These findings suggest that cellular responses to glycation and resulting stiffness depend on both the concentration of reducing sugar and the time spent under those conditions.

## 1. Introduction

Glycation, also referred to as non-enzymatic glycosylation, is a complex and spontaneous process in which reducing sugars (e.g., glucose, galactose, fructose, or ribose) react with free amino groups, typically lysine or arginine, to form reactive Schiff bases that are then transformed into more stable Amadori products. The Amadori products undergo further reactions to form advanced glycation end products (AGEs), which, as numerous studies have shown, are associated with the development or exacerbation of various diseases such as hypertension, atherosclerosis, Alzheimer’s disease, diabetes, and cancer [1,2]. AGEs can form covalent cross-links with proteins such as collagen and elastin of the extracellular matrix (ECM), which in turn results in increased tissue stiffness and resistance to enzymatic degradation [3]. These alterations in ECM stiffness can influence the expression of genes associated with mechanotransduction and contribute to various pathological conditions, such as inflammation, fibrosis, circadian clock impairment, stem cell aging, etc. [4]. It has been shown that mesenchymal stem cells cultured on soft matrices that mimic brain and stiffer matrices that mimic muscle or bone start expressing organ-specific transcription factors and undergo tissue-specific cell fate switches into neurons, myoblasts and osteoblasts, respectively [5,6]. AGE cross-linking has been implicated in vascular and articular cartilage stiffening, closely associated with the progression of arteriosclerosis and osteoarthritis, respectively [7,8]. Moreover, AGEs bind to the receptors for the AGEs (RAGEs), triggering downstream inflammatory cascade events and oxidative stress, exacerbating pathological processes [9]. The accumulation of adducts and AGEs with aging has been found in many tissues and organs, including the blood, blood vessel walls, kidneys, brain, peripheral nerves, joints, and skin [10,11,12,13]. To date, the impact of glycation on the aging process is undeniable. However, our understanding of the molecular mechanisms underlying cellular responses to glycation and subsequent changes in the mechanical properties of ECM remains incomplete.

The aim of this study was to investigate the effect of glycation on the gene expression profiles of human dermal fibroblasts (HdFbs (d75)) at the 12th (considered “young”) and 20th (considered “senescent”) passages cultured for 10 and 17 days in three-dimensional (3D) post-glycated collagen matrices. Collagen is a predominant component of ECM and, due to its long biological half-life, is susceptible to pathological non-enzymatic modifications, including glycation. Ribose was used as a glycating agent due to its higher reactivity compared to other reducing sugars [14].

## 2. Results

### 2.1. The Influence of D-Ribose Concentration on the Viability of Human Dermal Fibroblasts

**Figure 1:**
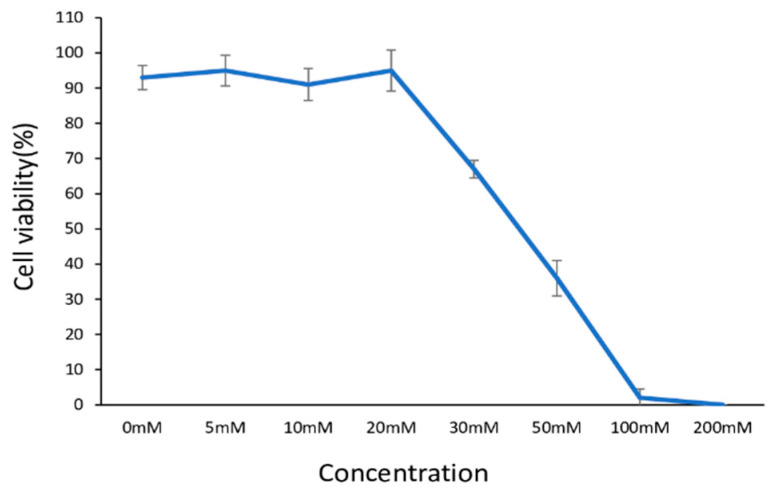
Dose-dependent viability of human dermal fibroblasts (HdFbs (d75)) treated with D-ribose. Viability was assessed by live/dead staining. Each data point represents a mean of 5 replicates ± SD.

To assess the effect of D-ribose on cell viability, HdFb (d75) cells at the 15th passage were cultured in glycated collagen hydrogels and stained with calcein-AM, propidium iodide, and Hoechst 33342 after 10 days of cultivation. The live/dead staining showed that when using ribose at concentrations below 20 mM, cell viability was unaffected and remained above 90% (Figure 1). Further increases in concentration led to a dose-dependent decrease in cell viability. Increasing the ribose concentration to 30 mM resulted in a decrease in cell viability of up to 65%. We determined that the IC50 for D-ribose is 43.2 mM. For further experiments, 15 mM and 30 mM were chosen.

### 2.2. Formation of Fluorescent AGEs in Post-Glycated Collagen Matrices

**Figure 2:**
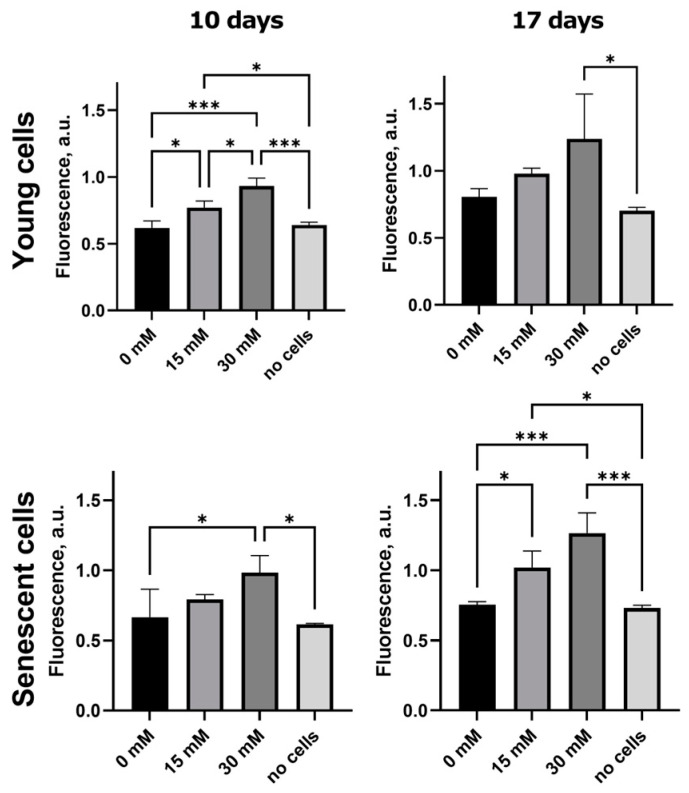
Formation of fluorescent advanced glycation end products (AGEs) in 3D post-glycated collagen matrices. The statistical processing between all groups (control was matrices with cells at 0 mM ribose) was performed with an ANOVA with a Bonferroni post hoc test, * *p* < 0.05, *** *p* < 0.001. All experiments were performed with three biological replicates.

It is known that the majority of identified AGEs have fluorescent properties [15]. To assess the formation of AGEs, we measured the autofluorescence intensity of gels with young and senescent cells, as well as gels without cells as a control. The glycated gels were prepared as described above using D-ribose at 15 mM and 30 mM concentrations. As shown in Figure 2, there were no statistically significant differences in autofluorescence intensity between non-cellular matrices and matrices with cells at 0 mM ribose. The content of fluorescent AGEs increased depending on the concentration of ribose in both gels with senescent fibroblasts and gels with young fibroblasts. Moreover, the intensity of autofluorescence increased depending on the cultivation time. This suggests that the accumulation of AGEs continued despite the fact that ribose treatment was only for the first 5 days. The peak of autofluorescence was observed at 30 mM ribose on the 17th day of cultivation.

### 2.3. Ribosylation Alters the Mechanical Characteristics of the Collagen Matrices

**Figure 3:**
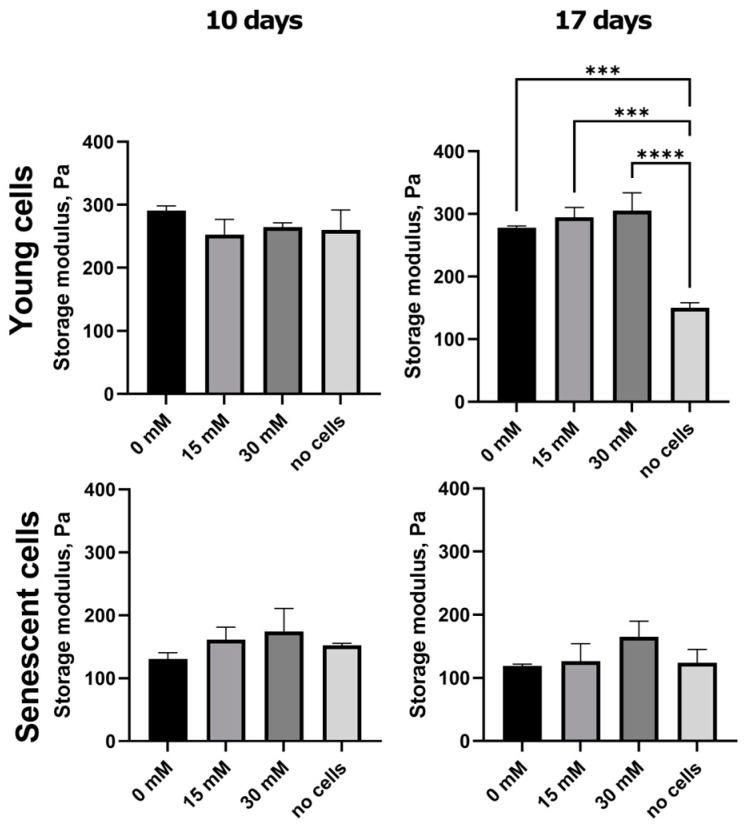
Storage modulus of collagen gels at 1 Hz frequency. *** *p* < 0.001, **** *p* < 0.0001.

Rheological measurements were performed to examine the mechanical changes in post-glycated matrices. Our results showed the trend of the stiffness of the matrix increasing depending on the concentration of ribose, although not statistically significantly (Figure 3). A significant difference was observed only in the gels containing young cells on day 17. There were no substantial differences between the gels at 10 and 17 days.

### 2.4. Transcriptome Analysis

Next, we performed RNA-Seq to analyze the effect of D-ribose, as a glycating agent, on gene expression profiles in normal fibroblasts at different stages of senescence. We used HdFb (d75) cells at the 12th and 20th passages cultured for 10 and 17 days in 3D post-glycated collagen matrices. According to the differential expression analysis, 15 mM ribose caused more than a two-fold change in expression (|LogFC| ≥ 1, LogCPM > 1, FDR  <  0.05) of 36 genes in the young cells and 772 genes in senescent cells. The effect of 30 mM ribose was more dramatic: 556 differentially expressed (DE) genes in young cells and 1396 DE genes in senescent cells were detected. We also compared the obtained lists of DE genes with the core matrisome (*n* = 274) and matrisome-associated genes (*n* = 753) downloaded from MatrisomeDB (https://matrisomedb.org, accessed on 23 March 2024). Lists of DE genes for all experimental variants, with information about genes belonging to particular categories of matrisome genes (collagens, proteoglycans, ECM glycoproteins, ECM-affiliated proteins, ECM regulators, and secreted factors), are presented in Table S1.

**Figure 4:**
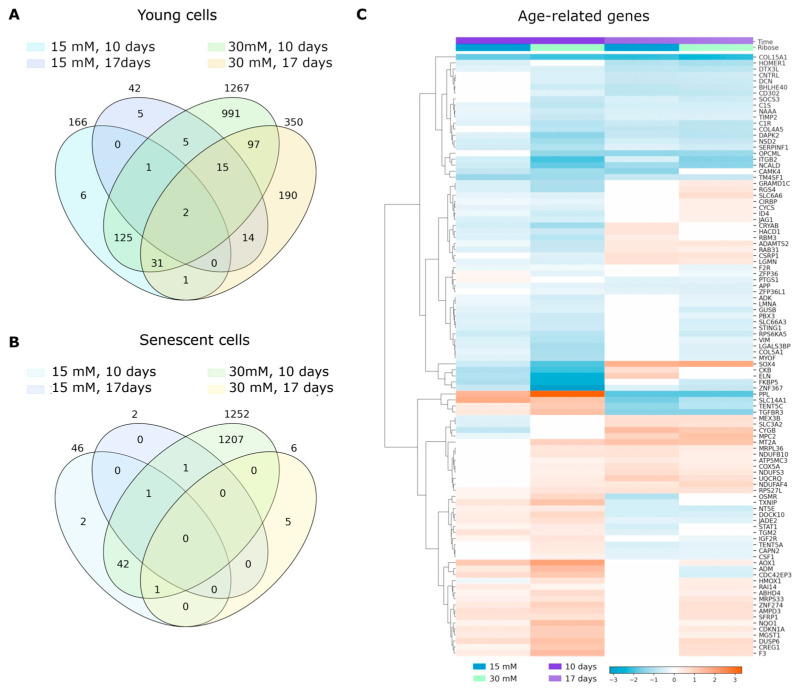
Differentially expressed genes. (**A**) Venn diagram showing the overlap of differentially expressed (DE) genes (|LogFC | ≥ 1, LogCPM > 0.5, FDR  <  0.05) in young human dermal fibroblasts (HdFbs) cultured in 3D post-glycated collagen matrices. (**B**) Venn diagram showing the overlap of DE genes (|LogFC | ≥ 1, LogCPM > 0.5, FDR  <  0.05) in senescent HdFbs cultured in 3D post-glycated collagen matrices. (**C**) Heatmap showing differentially expressed age-related genes in young cells cultured in post-glycated matrices. DE genes found in at least three comparisons are presented.

In cells of both passages, we detected an increase in the mRNA levels of the known senescence marker *CDKN1A*, key senescence-associated secretory phenotype (SASP) components (*CXCL8*, *CXCL 2*, *TGFB2*, *FGF2*, *VEGFB*), and a significant decrease in expression of the *LMNB1* and *EZH2* genes. These findings indicate that senescence is induced in fibroblasts cultured in a glycated matrix. We compared our lists of DE genes with human genes presented in the GenAge database [16], a database of genes related to longevity and/or aging in model organisms and aging-related human genes. As shown in Figure 4C, the changes in gene expression patterns were dependent on the concentration of ribose. Interestingly, the direction (increase or decrease) of expression for a number of genes changed over time, suggesting temporary regulation of cellular responses to the glycation of the microenvironment.

Increasing the ribose concentration to 30 mM resulted in changes in the expression of genes belonging to MMPs (*MMP3*, *MMP11*, *MMP12*, *MMP19*, and *MMP15*), the adamalysins (*ADAM33*, *ADAM23*, *ADAMTS3*, *ADAMTS7*, *ADAMTS14*, and *ADAMTS13*), the cathepsins (*CTSL*, *CTSS*, and *CTSB*), the semaphorins (*SEMA3D*, *SEMA3E*, *SEMA4B*, *SEMA4A*, and *SEMA7A*), the laminins (*LAMA2*), and the netrins (*NTNG1*, *NTN4*, and *NTN1*). It is worth noting that not only were the number of genes greater, but the changes in expression level were also stronger. For example, the *MFAP5* gene, encoding Microfibril-Associated Protein 5, showed an eight-fold increase in expression when treated with 30 mM ribose, whereas when treated with 15 mM ribose the change was two-fold. In Table 1, we present 13 common genes (|LogFC | ≥ 1, LogCPM > 1, FDR  <  0.05) that changed their expression in all experimental variants on day 10 of culture. Among them, there are four matrisome genes: *COL11A1*, *MMP3*, *EPGN*, and *HAPLN1*.

Overall, our results revealed that the number of DE genes was lower in both 0 Mm vs. 15 mM and 0 vs. 30 mM comparisons after day 17 of culture compared to day 10 (Figure 4A,B). This might be due to cell stabilization and adaptation into the glycated 3D gels with time. According to the literature data, the response of cells to changes in the mechanical microenvironment depends on both the magnitude and timescale of exposure [9]. Among the top upregulated genes on day 17 compared to day 10, we can distinguish the *ANKRD1*, *KRT14*, *CDCA3*, *KIF20A*, and *TMSB15A* genes with LogFC values < −2.44 in senescent cells, and *TTR*, *ACAN*, *RAMP1*, *CLIC3*, *ANKRD1* with LogFC values < −3.88 in young cells cultured in the 15 mM ribose glycated matrices. At 30 mM, the highest FC values in senescent cells were found for *TROAP*, *KIF20A*, *MKI67*, *WFDC1*, and *IQGAP3* (LogFC values < −5.53), and found for *IQGAP3*, *ARC*, *HAPLN1*, *KIF20A*, *DLGAP5* (LogFC values < −5) in young cells. Up- and downregulated genes (|LogFC | ≥ 1, LogCPM > 1, FDR  <  0.05) on day 17 compared to day 10 in all conditions (with and without ribose) are listed in Table S2.

**Figure 5:**
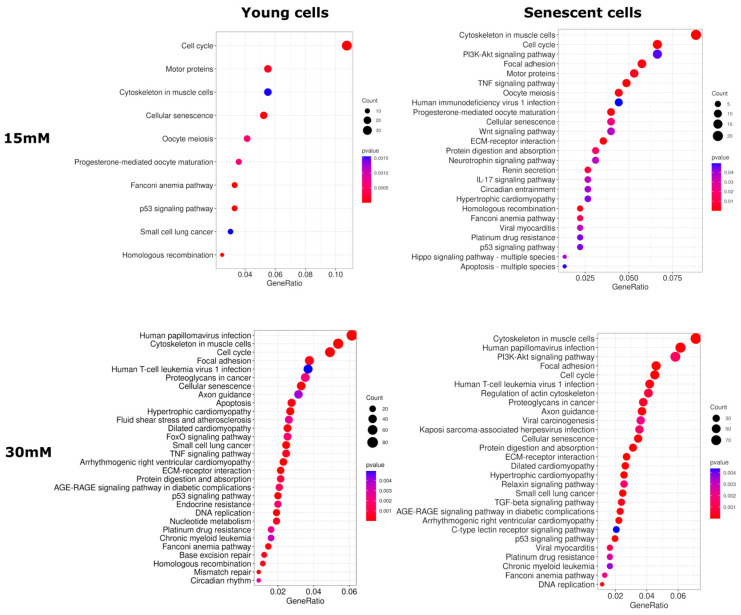
KEGG pathways modulated by glycation in HdFbs on the 10th day of cultivation. The *X*-axis and dot size indicate the gene ratio (the number of DE genes involved in the KEGG pathway divided by the total number of genes that are annotated as participants of this pathway).

Next, to determine which cellular processes were affected by the post-glycation of collagen matrices, we performed a Kyoto Encyclopedia of Genes and Genomes (KEGG) pathway enrichment analysis using lists of DE genes. According to the results, the DE genes belonging to KEGG pathways such as «Cytoskeleton in muscle cells», «Focal adhesion», «Platinum drug resistance», «ECM-receptor interaction», «PI3K-Akt signaling pathway», «Cell cycle», «Protein digestion and absorption», «Cellular senescence», «p53 signaling pathway», and «Fanconi anemia pathway» were statistically significantly enriched in all comparisons on day 10 of culture. On day 17, common KEGG pathways included «Human papillomavirus infection», «Cytoskeleton in muscle cells», «PI3K-Akt signaling pathway», and «Retrograde endocannabinoid signaling». The lists of enriched KEGG pathways altered by glycation are presented in Figure 5 and Figure S1.

## 3. Discussion

In this study, we describe transcriptomic changes in young and senescent cells during culture in glycated 3D collagen matrices. Our results revealed that non-enzymatic glycation with 30 mM ribose resulted in broader transcriptome response compared to 15 mM ribose treatment. These data are consistent with the results of autofluorescence and rheological analysis showing that AGE accumulation and matrix stiffness also increase in a dose-dependent manner. AGEs are known to accumulate with age in collagen-rich tissues, where they can form cross-links and thereby increase tissue stiffness [17]. Normally, cells sense and regulate ECM mechanical properties by maintaining a balance between matrix formation and degradation to maintain tissue homeostasis [18]. Dysregulation of ECM remodeling can lead to fibrosis, a decrease in regenerative potential, and inflammatory processes, as well as other pathologies that are characteristic of aging tissues [19]. It has been shown that glycation cross-linked fibers have a >5 times higher resistance to enzymatic degradation [20]. Our results showed significant changes in the expression of genes encoding MMPs, which are key ECM remodeling enzymes responsible for the degradation of various extracellular proteins [21]. A significant increase in expression of *MMP3* gene was found in both young and senescent cells in response to ribose addition. MMP-3, also known as stromelysin-1, catalyzes the degradation of several substrates, including cartilage proteoglycan, collagen types II, IV, IX, and XI, laminin, and fibronectin, and is closely related to osteoarthritis severity [22]. Osteoarthritis is a disease characterized, among other factors, by the age-related accumulation of AGEs in articular cartilage [23]. Another member of the MMP family, *MMP15*, which has also been previously shown to be associated with osteoarthritis, showed increased gene expression in senescent cells [24]. The *MMP19* and *MMP11* genes were downregulated in fibroblasts of both passages. The activity of MMPs is regulated by endogenous tissue inhibitors of metalloproteinases (TIMPs), although in this study we were unable to detect genes encoding TIMPs among DE genes past significance (FDR < 0.05) and 2-fold differential expression thresholds. On the other hand, it has been shown that in skin, an increase in MMP level may not be accompanied by a corresponding increase in TIMP level, thereby accelerating collagen fragmentation and, correspondingly, skin aging [25,26].

Among other DE genes related to the category of ECM regulators, we can highlight genes encoding adamalysins and cathepsins, proteases that not only play an important role in ECM remodeling but are also implicated in a wide range of cellular activities [27,28]. A number of ECM-affiliated genes that were modulated by 30 mM ribose were linked to the semaphoring–plexin system. Semaphorins and plexins are ligands and cell surface receptors that provide guidance cues for migration and are implicated in cytoskeletal reorganization, adhesion, and cell proliferation [29,30].

Increased matrix stiffness predictably affected the expression of many core matrisome genes. Most DE genes encoding collagens showed decreased expression in glycated matrices. Interestingly, a similar collagen expression profile is characteristic of the skin of elderly individuals [31]. Our results are partially consistent with Li et al. (2021), who detected age-associated changes in the expression of COL21A1, COL12A1, COL4A1, HAPLN3, ACAN, and GPC4 [32]. We found dose- and passage-dependent decreases in gene expression of the hyaluronic acid-binding proteoglycans, such as *ACAN* and *HAPLN3*. These proteoglycans are closely associated with viscoelasticity and hydration, properties essential for healthy skin [33]. Among the genes encoding glypicans, *GPC4*, *GPC3* and *GPC6* also underwent significant changes in expression. Glypicans are cell-surface heparan sulfate proteoglycans that regulate several important signaling pathways, such as the Wnt, Hedgehog and BMP signaling pathways [34]. Indeed, among the enriched KEGG pathways, we found the Wnt signaling pathway, as well as several other signaling pathways, that had previously been shown to be associated with glycation and aging. It has been shown, for example, that AGEs can promote the calcification of human arterial smooth muscle cells through the regulation of PI3K/AKT-GSK3-ββ signaling [35]. The TGF-beta signaling pathway, which is known to be activated during aging [36] and is closely related to fibrosis [37], was highly enriched with DE genes.

Senescent cells are known to secrete growth factors, interleukins, chemokines, and proteases, which form the SASP. Our results indicate that glycation enhances SASP in a dose-dependent manner, including in young cells. In addition, we found that glycation led to changes in the expression of many age-related genes (according to the GenAge database) [16]. Indeed, stochastic non-enzymatic modifications of ECM are closely associated with hallmarks of aging, including cellular senescence, and can be considered key targets for anti-aging interventions [3]. Interestingly, during long-term cell culture in glycated matrices, the expression of SASP genes was less pronounced, which may be related to the temporal regulation of SASP [38]. Some SASP factors are secreted at different times depending on culture conditions and cell type. It has been shown, for example, that the induction of senescence by UVB in keratinocytes led to increased mRNA levels and secretion of IL-6 and IL-8 at day 3, which disappeared on day 7 [39]. Analysis of time-series gene expression profiles of replicative senescence in HdFbs has revealed the presence of several phases of SASP gene expression [40]. The initial phase is characterized by overexpression of many interleukins and chemokine ligands, while the later phases are characterized by differential expression of genes associated with extracellular matrix remodeling, such as MMPs [40].

Overall, the results obtained indicate that glycation-induced changes in transcriptome profiles were greater (both gene numbers and FC values) on day 10 of cultivation compared to day 17, while matrix stiffness values were comparable. For example, after 10 days of culture, there was a five-fold decrease in the expression of the *ELN* gene encoding elastin, whereas on the 17th day of culture, no changes in the expression of this gene were detected. We also detected a decrease in gene expression levels of the glycoproteins fibulin (*FBLN7*) and fibrillin (*FBN1*, *FBN2*), which in most tissues are associated with elastin to form functional elastic fibers [41]. A loss of elastic fiber integrity reduces the extensibility and resilience of tissue as well as the ability of cells to mechanosense the mechanical state of tissue [42]. Moreover, the results obtained for the first time point (10 days) included, among others, genes encoding the transmembrane receptor integrins (ITGA and ITGB genes), the downstream linker proteins alpha-actinin (*ACTN1*) and talin (*TLN2*), the Rho family of GTPases (*RHOA*, *RHOA*), actin (*ACTB*), actin-related proteins (*ACTR2*, *ACTR5*), and non-muscle myosins (*MYH10*, *MYH9*), which are key players in the reorganization of the actin cytoskeleton underlying many cellular responses to mechanical stress [43]. Since the Rho/ROCK signaling pathway is a key regulator of the cellular response to changes in ECM stiffness, the use of Rho kinase inhibitors and its downstream targets appears to be a promising anti-aging strategy [44]. Other potential anti-aging interventions include the application of bioactive compounds with anti-glycation properties (chebulic acid, quercetin, fructosamine-3-kinase, or fructosyl-amino acid oxidase) [45,46,47,48], agents that stimulate elastogenesis, RAGE antagonists, soluble RAGE [49], and breakers of glucosepan, the major cross-link of the senescent ECM [50]. It is worth noting that we did not observe statistically significant changes in the expression of *AGER*, the gene encoding RAGE, on either the 10th or 17th day of cultivation. This may be attributed to the fact that we ceased adding the glycating agent on the 5th day. On the other hand, the enrichment analysis revealed a statistically significant enrichment of the “AGE-RAGE signaling pathway in diabetic complications” KEGG pathway, which indicates a change in the expression of genes involved in this pathway. RAGE engagement by AGEs activates intracellular signaling pathways including stimulation of NADPH oxidase, mitogen-activated protein kinase (MAPK) cascades, and the PI3K/AKT, JAK/STAT, and Cdc42/Rac1 pathways followed by the induction of transcription factors NF-κB and AP-1 [51]. These pleiotropic transcription factors regulate the expression of cytokines, growth factors, metalloproteinases, and adhesion molecules, many of which showed differential expression in our experiment.

Taken together, the current study demonstrated that D-ribose, as a glycating agent, increases the formation of glycation end products and the stiffness of collagen gels in a dose-dependent manner, which is consistent with the generally accepted concept of the effect of ECM glycation on the mechanical and functional properties of tissues. At the molecular level, the glycation of collagen matrices caused significant changes in the gene expression profile in both young and senescent fibroblasts, including the modulation of many age-associated genes and signaling pathways. Furthermore, our findings revealed that the gene expression patterns of cells cultured in post-glycated 3D matrices can significantly vary over time, occasionally in the opposite direction. This suggests the temporal regulation of the cellular response to the glycated microenvironment. The results obtained may have important implications for current understanding of the cell–matrix interaction under glycation conditions.

## 4. Materials and Methods

### 4.1. Cell Culture

Human dermal fibroblasts (HdFbs (d75)) were obtained from the Cell Culture Collection of N.K. Koltzov Institute of Developmental Biology, Moscow, Russia. Cells were cultured in EMEM medium with L-glutamine and double amino acids (Biolot, Saint-Petersburg, Russia) supplemented with 10% fetal bovine serum (Thermo Fisher Scientific, Waltham, MA, USA) and 1% penicillin/streptomycin (Thermo Fisher Scientific, Waltham, MA, USA) in a CO_2_ incubator at +37 °C, 5% CO_2_. Cell culture medium was changed every three days. Cells were detached using TrypLE™ Express (Thermo Fisher Scientific, Waltham, MA, USA) after reaching approximately 80% of confluence. Cells of the 12th (considered “young”), 15th, and 20th (considered “senescent”) passages were chosen for further experiments.

### 4.2. Formation of Collagen Gels and Glycation Process Using Ribose (Ribosylation)

For the preparation of collagen gels, a homogeneous aqueous solution of concentrated collagen type I Viscoll (80 mg/mL) (Imtek Ltd., Moscow, Russia) was diluted in 2 mM acetic acid to a concentration of 10 mg/mL. Then, the collagen solution was neutralized with 0.2 N NaOH (Sigma-Aldrich, St Louis, MO, USA) and mixed with the culture medium, resulting in a neutralized homogenous 5 mg/mL collagen solution. All manipulations were made at +10 °C to avoid premature polymerization of collagen hydrogels. The resulting collagen solution was carefully mixed with the suspension of cells and distributed into pre-chilled plates and preincubated for 1 h for polymerization of collagen.

The glycated gels were prepared using a non-enzymatic post-glycation method [52]. D-Ribose (Sigma-Aldrich, USA) was used as a reducing sugar to obtain the glycated gels. According to the literature data, the ability of ribose to modify the physical and chemical properties of type I collagen gels is higher than that of glucose [53,54]. After gel polymerization, the medium was replaced with the same medium supplemented with ribose. Cell-laden 3D hydrogels were treated with different doses of ribose for 5 days to ensure cross-linking between the collagen and the reducing sugar. Then, a medium without ribose was used, which was changed every 2–3 days.

### 4.3. Viability Assay

The viability of the HdFbs cultured in 3D glycated collagen matrices was determined using calcein-AM/propidium iodide/Hoechst staining. Cells of the 15th passage were mixed with the collagen before polymerization of the gel. After polymerization of the matrices, various concentrations of D-ribose (0 mM, 10 mM, 15 mM, 20 mM, 30 mM, 50 mM, and 100 mM) were added to the medium for 5 days. Cell viability was assessed on the 10th day of culture. Staining solution was prepared using a culture medium containing calcein AM (Thermo Fisher Scientific, USA), propidium iodide (Sigma-Aldrich, USA), and Hoechst 33342 (Thermo Fisher Scientific, USA). The medium was aspirated from the wells, replaced with the staining solution, and incubated for 30 min before imaging. Images were taken using an Evos M7000 (Thermo Fisher Scientific, USA). Three images were obtained from each well (at least 20 slices for each Z-stack). Cell viability was calculated by counting the number of live cells divided by the total cell number. Images were analyzed using Image J software (v 1.53j) with the default parameters [55]. 

### 4.4. Autofluorescence Analysis

The level of fluorescent AGEs in post-glycated matrices by 15 mM and 30 mM ribose were measured on the 10th and 17th days of culture. Measurements were carried out for both gels with cells and gels without cells. The gels were extensively washed from the medium in an excess amount of distilled water for 2 days on a shaker with several water changes. Autofluorescence of the gels was determined using a plate spectrofluorimeter Victor Nivo (PerkinElmer, Waltham, MA, USA) with an excitation filter of 355/40 nm and an emission filter of 450/10 nm. At least three gels were measured per condition; the statistical processing was performed with an ANOVA with a Bonferroni post hoc test in Graphpad Prism v. 9.4.1.681 (GraphPad Software, San Diego, CA, USA).

### 4.5. Rheology

The rheological properties of the collagen gels were characterized with a Physica MCR 302 rheometer (Anton Paar, Graz, Austria) controlled by the RheoCompass software (v1.25.373) with a plate–plate geometry (25 mm diameter) at a controlled temperature of 25 °C in a humid camera. The gels were transferred from 12-well plates to the rheometer stage right before the measurements. The oscillatory strain sweep test was performed at a fixed frequency of 1 Hz to determine the linear viscoelastic region (LVR), and the frequency sweep test was performed for the viscoelastic characterization. The tests were repeated at least three times, the storage modulus, G′ (Pa), and the loss modulus, G″ (Pa), were measured. For the strain sweep test, 15 points were chosen with a range between 0.05 and 10% of the sweep amplitude. The frequency sweep test was then performed at a 0.1% strain amplitude that was within the LVR of each gel type; 16 points were chosen within the logarithmically spaced frequency range between 0.1 and 30 Hz.

### 4.6. Preparation of cDNA Libraries and Sequencing

For transcriptomic analysis, total RNA from cells of the 12th and 20th passages was isolated using a Quick-RNA MiniPrep Kit (Zymo Research, Irvine, CA, USA) according to the manufacturer’s instructions. The gels with cells were pre-treated with Collagenase Type II (Gibco, Waltham, MA, USA) at a concentration of 150 U/mL and kept in a CO_2_ incubator until the collagen had visually dissolved (approximately 40 min). The quantity and quality of total RNA were checked using a Qubit^®^2.0 Fluorometer (Thermo Fisher Scientific, USA), NanoDrop^®^ ND-1000 spectrophotometer (NanoDrop Technologies Inc., Wilmington, DE, USA), and Agilent 2100 Bioanalyzer (Agilent Technologies, Santa Clara, CA, USA). For subsequent library preparation, samples with a RIN (RNA Integrity Number) > 8 were used. To obtain mRNA, total RNA was enriched by Poly (A) using VAHTS mRNA Capture Beads (Vazyme Biotech, Nanjing, China). Double-stranded cDNA libraries were prepared using a VAHTS^®^ Universal V8 RNA-seq Library Prep Kit for Illumina (Vazyme Biotech, Nanjing, China) according to the manufacturer’s protocol. cDNA quality was checked with the bioanalyzer Agilent 2100 using a High Sensitivity DNA Chip (Agilent Technologies, USA). Then, cDNA libraries were normalized to 4 nM, pooled together, and sequenced with 100 bp single-end reads on the NextSeq2000 System (Illumina, San Diego, CA, USA). The sequencing data are available at the NCBI Sequence Read Archive (GSE293225).

### 4.7. Bioinformatics Analysis

For the obtained RNA-Seq data in the fastqc format, quality assessment was performed using FastqQC and MultiQC (https://www.bioinformatics.babraham.ac.uk/projects/fastqc/, accessed on 23 March 2025). The Trimmomatic tool was used to remove bad-quality reads and adapter sequences from RNA-Seq data, which was followed by mapping to the reference genome (GRCh38 assembly) using the STAR tool v2.7.11b [56,57]. FeatureCounts (Subread package v.1.6.4, Parkville, Australia) was used to calculate the read counts per gene [58]. The analysis of differential gene expression was performed in the R statistical environment using the edgeR 3.36 package [59]. The TMM (Trimmed Mean of M-values) method was used to normalize the data. In the analysis of differential gene expression, the following quasi-likelihood F-test (QLF test) and the non-parametric Mann–Whitney test was applied (U-test). The Benjamini–Hochberg correction was applied to calculate the false positive rate (FDR). Gene set enrichment analysis (GSEA) was performed using the clusterProfiler package (v.3.14.3, Guangzhou, China) with the Kyoto Encyclopedia of Genes and Genomes (KEGG) database [60].

## Figures and Tables

**Table 1:** Differentially expressed genes in dermal fibroblasts on day 10 of cultivation in 3D glycated collagen matrices.

Gene Symbol	Young Cells		Senescent Cells	
	LogFC, 15 mMR	LogFC, 30 mMR	LogFC, 15 mMR	LogFC, 30 mMR
*AP005018.2*	−1.17	−2.5	−1.31	−2.66
*COL11A1*	−1.72	−2.93	−1.65	−2.81
*WFDC1*	−1.8	−3.69	−1.59	−5.35
*TMEM158*	1.08	3.03	1.44	4.16
*GRIK2*	−1.23	−2.83	−1.12	−2.96
*GTSE1*	−1.38	−4.26	−1.1	−2.24
*CDCA3*	−1.03	−3.65	−1.07	−2.97
*PHACTR3*	−1.12	−3.19	−1.01	−3.87
*EPGN*	1.65	2.86	1.06	1.95
*KIF20A*	−1.27	−6.07	−1.01	−5.65
*HAPLN1*	−1.68	−4.32	−1.66	−3.33
*LINC00578*	−1.36	−2.43	−1.41	−4.7
*MMP3*	1.15	3.89	1.37	5.67

LogCPM > 1, FDR  <  0.05.

## Data Availability

The obtained RNA-Seq data are available at the NCBI Sequence Read Archive (GSE293225).

## Supplementary Materials

The following supporting information can be downloaded at: https://www.mdpi.com/article/10.3390/ijms26104769/s1.
