# Supplementary figures and images for: Changes in Gene Expression Patterns in Young and Senescent Fibroblasts in Glycated Three-Dimensional Collagen Matrices
Source: Int J Mol Sci. 2025 May 16;26(10):4769. doi: 10.3390/ijms26104769 (PMC12112436; doi:10.3390/ijms26104769)

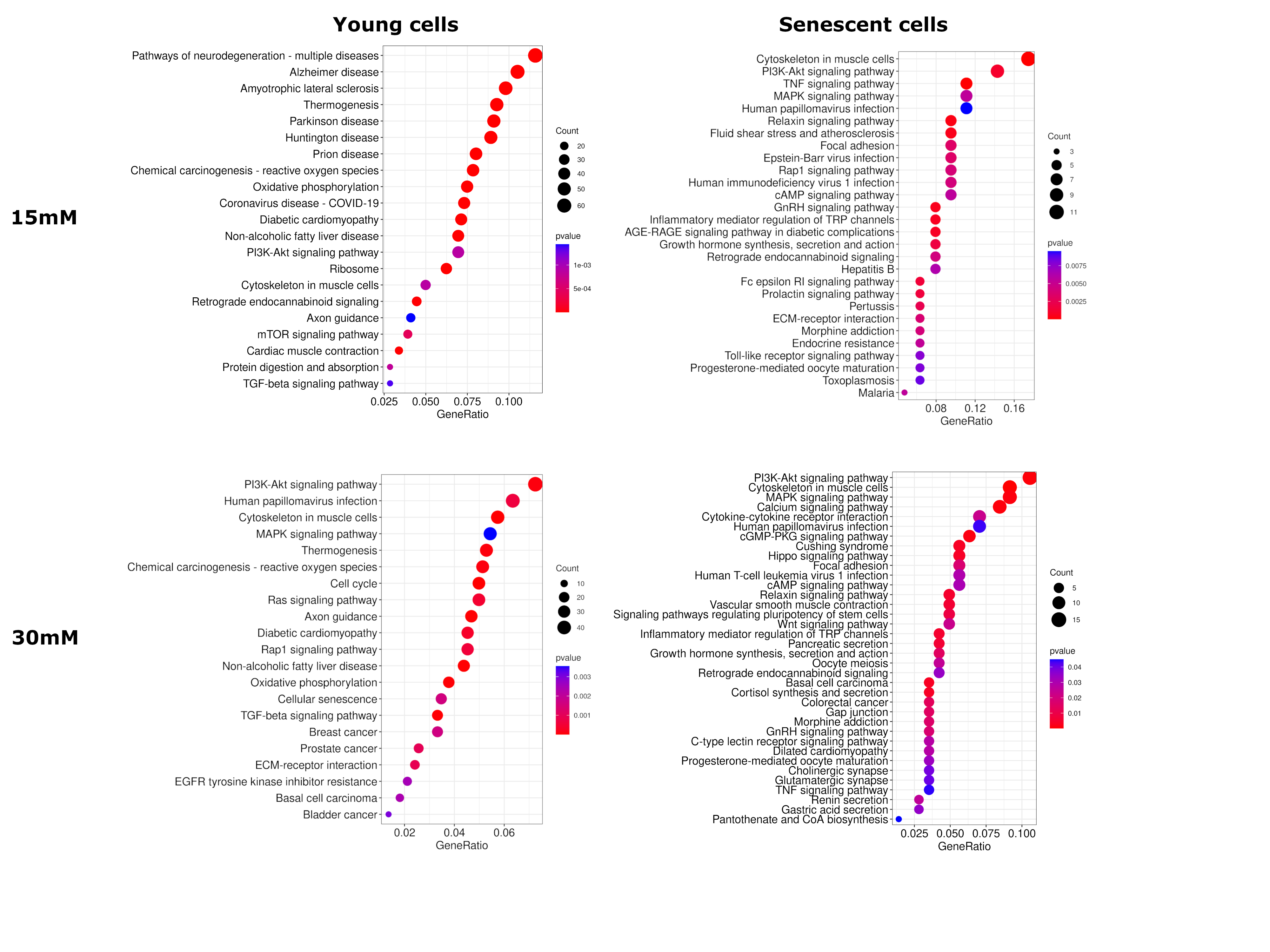

Supplement: Supplementary file 1 [file ijms-26-04769-s001.zip › Supplementary Figure 1.png]
